# Insights on the regulation of photosynthesis in pea leaves exposed to oscillating light

**DOI:** 10.1093/jxb/erac283

**Published:** 2022-08-29

**Authors:** Dušan Lazár, Yuxi Niu, Ladislav Nedbal

**Affiliations:** Department of Biophysics, Faculty of Science, Palacký University, Šlechtitelů 27, 783 71 Olomouc, Czech Republic; Institute of Bio- and Geosciences/Plant Sciences (IBG-2), Forschungszentrum Jülich, Wilhelm-Johnen-Straße, D-52428 Jülich, Germany; Department of Biophysics, Faculty of Science, Palacký University, Šlechtitelů 27, 783 71 Olomouc, Czech Republic; University of Essex, UK

**Keywords:** Fluctuating light, forced oscillations, pea, photosynthesis, photosystem I and II, *Pisum sativum*, proton motive force, regulation

## Abstract

Plants growing in nature often experience fluctuating irradiance. However, in the laboratory, the dynamics of photosynthesis are usually explored by instantaneously exposing dark-adapted plants to constant light and examining the dark-to-light transition, which is a poor approximation of natural phenomena. With the aim creating a better approximation, we exposed leaves of pea (*Pisum sativum*) to oscillating light and measured changes in the functioning of PSI and PSII, and of the proton motive force at the thylakoid membrane. We found that the dynamics depended on the oscillation period, revealing information about the underlying regulatory networks. As demonstrated for a selected oscillation period of 60 s, the regulation tries to keep the reaction centers of PSI and PSII open. We present an evaluation of the data obtained, and discuss the involvement of particular processes in the regulation of photosynthesis. The forced oscillations provided an information-rich fingerprint of complex regulatory networks. We expect future progress in understanding these networks from experiments involving chemical interventions and plant mutants, and by using mathematical modeling and systems identification and control tools.

## Teaser

Measurement of photosynthetic signals in pea leaves exposed to sine-function oscillations of light provides information about the regulation of photosynthesis in fluctuating light.

## Introduction

Adaptation of the photosynthetic apparatus to contrasting and often extreme environmental conditions, including dynamically changing light intensity (e.g. Yamori, 2016), has been one of the essential diversifying factors in the evolution of plants. To deal with a changing environment, plants have developed elaborate and diverse regulation systems (e.g. Tikhonov, 2015) to optimize yields and to minimize potential damage by harmful by-products (e.g. Pospíšil, 2016). Increasing our understanding of the dynamics of photosynthetic regulation in nature requires techniques to investigate plants in fluctuating light (e.g. Rascher and Nedbal, 2006; Matsubara, 2018; Gjindali *et al.*, 2021). Simulations of the natural random fluctuations in light, and approximations such as by repeated step-changes in light intensity, have been appearing in the literature with growing frequency (e.g. Cruz *et al.*, 2016; Annunziata *et al.*, 2017; Vialet-Chabrand *et al.*, 2017; Adachi *et al.*, 2019; Li *et al.*, 2019).

By analogy to the frequency-domain analyses widely used in physics and engineering, Nedbal and Březina (2002) introduced an alternative approach using regular, harmonically oscillating light. Any fluctuating light, random or regular, can be represented by a weighted sum of elemental harmonic components of characteristic frequencies (Schwartz, 2008), and thus, the complexity of such an investigation can be reduced by exposing plants to light oscillating sinusoidally with a single frequency, called harmonic modulation. The angular frequency of the modulation 2π/*T*, where *T* is the period of the light oscillation, can be gradually changed to cover the whole range of distinct dynamic components as they occur in a particular natural environment. Nedbal *et al*. (2003, 2005) have already applied such a frequency-domain analysis in plant research and, more recently, Nedbal and Lazár (2021) have supported it with mathematical models and further experiments. Since the course of the measured photosynthetic signal forced by the sinusoidal illumination deviates from the course of illumination, the response of the measured signal to illumination is non-linear.

On the other hand, intrinsic molecular reactions occurring in the photosynthetic system might also be non-linear; most often, this is when the rate of change of the system variable also depends on other system variables. From that point of view, the intrinsic non-linearity can be constitutive, i.e. directly related to the primary functions of the explored system, and it can be regulatory non-linearity (Bich *et al.*, 2016). An example of constitutive non-linearity is the cyclic electron transport (CET), where the rate of change of reduced plastoquinone also depends on reduced ferredoxin, which donates electrons to plastoquinone. On the other hand, a typical example of regulatory non-linearity is the non-photochemical quenching (NPQ) of chlorophyll fluorescence (ChlF). The intrinsic non-linearities manifest themselves as the presence of the so-called upper harmonics (multiplies of the basal frequency of modulation, i.e. 2 × 2π/*T*, 3 × 2π/*T*, etc.) in the measured signal (Nedbal and Lazár, 2021); in other words, if the measured signal is not saturated, additional waves and/or bumps appear in the signal. The intrinsic non-linearities might result in a non-linear dependence of the measured output photosynthetic signal on the system input signal (light illumination). The harmonically oscillating illumination thus yields insights into the fundamental dynamics of photosynthesis in fluctuating light and reveals frequency domains that characterize various mechanisms of photosynthesis regulation (Nedbal and Březina, 2002; Nedbal *et al*., 2003, 2005; Nedbal and Lazár, 2021).

The response of plants to changing light is often sensed by measuring ChlF (reviewed in Bąba *et al.*, 2019). The main part of variable ChlF in the initial rise of the ChlF induction that occurs with the exposure of dark-acclimated plants to constant light is attributed to the progressing photochemical reduction of the first quinone electron acceptor, Q_A_, of PSII (reviewed in Lazár, 1999; Stirbet and Govindjee, 2012). However, even when Q_A_ is already reduced, i.e. the reaction centers (RC) of PSII (RCII) are closed, the ChlF can further increase, and this is probably caused by light-induced conformational changes of PSII and/or the electric fields (Vredenberg and Bulychev, 2002; Magyar *et al.*, 2018; Laisk and Oja, 2020; Sipka *et al.*, 2021). This rise of ChlF accounts for about a third of the total measured with saturating light.

The photochemical alteration of ChlF also occurs in harmonically modulated light. Some of the RCIIs may open around the light minima and close around the light maxima. The respective fractions of open and closed RCIIs are expected to change with the frequency and amplitude of the harmonic light modulation, thereby providing important information about the dynamics of the primary processes and the regulation in fluctuating light.

Later phases of the ChlF induction are typically attributed to the activation of the CO_2_ assimilation in the Calvin–Benson cycle and to NPQ of ChlF. The NPQ is a generic term that, strictly speaking, also includes processes that decrease the photosynthetic energy conversion and the ChlF emission in an intense light by lowering the effective absorption cross-section of the RCIIs rather than by excitation quenching by heat dissipation. More typically, however, NPQ is attributed to the dissipation of the excess excitation energy as heat (Papageorgiou and Govindjee, 2014). In plants, this quickly reversible, ‘high-energy’ NPQ (qE) is related to acidification of lumen in light, and formation of carotenoid zeaxanthin and antheraxanthin from violaxanthin, and requires the presence of the PSII protein subunit S (reviewed in Holzwarth and Jahns, 2014; Ruban and Wilson, 2021).

It may be presumed that the Calvin–Benson cycle remains active in the harmonically oscillating light as long as the light minima are not too long and not too close to darkness. On the other hand, the NPQ-regulation is likely to modulate the ChlF emission response to an extent determined by the interference between light modulation frequency and amplitude on the one hand, and by the activation and deactivation characteristics of the NPQ processes on the other. We presume that this interference represents a unique opportunity to separate and identify the individual NPQ mechanisms dynamically.

Another tool to dissect the various contributions to ChlF modulation is the usage of multiple-turnover saturating pulses (MTSPs) that can transiently close the RCIIs by congesting the electron transport pathways. Time-domain measurements with constant light routinely adopt usage of MTSPs (reviewed in Lazár, 2015). However, it has to be kept in mind that the maximal ChlF determined by applying MTSPs might also reflect, to some extent, effect(s) that are not related to the photochemical closure of the RCIIs (see above).

The dynamics of the transmittance optical proxy I830 can complement the information obtained with the ChlF. The I830 signal, measured as the difference of the leaf transmittance at 875 nm and 830 nm, mainly reflects the redox dynamics of the primary electron donor P700 in PSI (e.g. Klughammer and Schreiber, 1991); however, redox changes of plastocyanin and ferredoxin might also contribute. Similar to ChlF, the application of MTSPs allows additional information to be obtained about PSI functioning (e.g. Schreiber and Klughammer, 2008a). The Walz Dual-PAM-100 instrument that we use in the present study can measure ChlF and the I830 signal simultaneously. The instrument can also provide another essential complementary insight into photosynthetic dynamics by measuring the difference in the leaf transmittance at 550 nm and 515 nm, named P515 (e.g. Schreiber and Klughammer, 2008b). This signal reflects the electrochromic shift of the absorption bands of carotenoids and Chl *b* caused by the electric potential difference, ∆*Ψ*, across the thylakoid membrane (TM). Other effects might contribute to the P515 signal, namely absorption changes at 505 nm (see Ruban and Wilson, 2021), 535 nm (see Ruban *et al.*, 2002), and 550 nm (see Van Wittenberghe *et al.*, 2019). Calculating the transmittance difference at the two wavelengths eliminates the absorption change at 535 nm. Changes in the baseline in time-scales different from changes in P515 distinguish the absorption changes at 505 nm and 550 nm (Schreiber and Klughammer, 2008b). Further enhancing the information obtained, the relaxation of the P515 signal that occurs when the actinic light is switched off can be used to separate and quantify the chemical (∆pH) and electric (∆Ψ) components of the proton motive force (PMF) over the TM (Cruz *et al.*, 2001; Schreiber and Klughammer, 2008b).

The application of MTSPs at different phase-points of the light oscillation, in the same way as routinely done during induction in the dark-to-light transition mentioned above, further enriches the information content of ChlF and I830 reporter signals (Nedbal *et al.*, 2003). Similarly, P515 relaxation in different phases of the light oscillation can also be recorded by abruptly switching off the actinic light.

In this study, we provide detailed information about the measurement of the forced oscillations in ChlF, I830, and P515 signals and the procedures for measuring the quantum yields and other parameters that we imposed by subjecting pea plants to oscillating light. We show the forced oscillations of the signals of the leaves caused by illumination with red actinic light oscillating with periods ranging from 1 s to 300 s. For a selected period of 60 s, we show quantum yields related to the functioning of PSII and PSI and changes of PMF and its ∆pH-dependent and ∆Ψ parts changing with the light oscillation, the latter presented for the first time. Further, we discuss an evaluation and analysis of some of the parameters. By comparing the evaluated parameters, we infer the mechanisms of the regulation of photosynthetic function in fluctuating light.

## Material and methods

### Plant material

Seeds of pea (*Pisum sativum*) were sown in pots with perlite and Knop solution and placed in a growth chamber (AR-100L3, Percival Scientific, USA) under controlled conditions of 16 h white light (150 μmol PAR photons m^−2^ s^−1^) and 8 h dark. The temperature was kept between 20–22 °C and the relative air humidity was 60% during germination and growth. Pots with 15–20-day-old plants were removed from the chamber and kept in darkness for 30 min before the measurements. One by one, well-developed green leaves attached to the plants were gently inserted between the optical heads of the instrument and measured.

### The Dual-PAM instrument, and light colors and intensities used

We used a Walz Dual-PAM-100 instrument with the optical heads DUAL-E and DUAL-DB for simultaneous measurements of chlorophyll fluorescence (ChlF) and the I830 signal on the same leaf. We measured the P515 reporter signal consecutively on another leaf using the DUAL-EP515 and DUAL-DP515 optical heads.

Red (635 nm) actinic light was generated by the Walz instrument with its intensity sinusoidally oscillating between 0–250 μmol photons m^−2^ s^−1^. We chose this intensity range since it led to the most pronounced non-linear upper-harmonic modulation (i.e. the measured output signal is described by a sum of sine functions whose periods, in addition to *T*, are *T*/2, *T*/3, … , where *T* is the period of input sinusoidal light) that is a hallmark of regulatory non-linearity (Nedbal and Březina, 2002; Bich *et al.*, 2016; Nedbal and Lazár, 2021). Blue measuring flashes (460 nm) were used to excite ChlF, which was detected by the pulse amplitude modulation (PAM) method. I830 and P515 signals were detected as the differences of the leaf transmittances at 875 nm and 830 nm, and at 550 nm and 515 nm, respectively. Multiple-turnover saturating pulses (MTSPs) of white light (intensity 5000 μmol PAR photons m^−2^ s^−1^, duration 0.3 s) and illumination by far-red light (720 nm, duration 9 s) were also generated by the optical heads of the instrument.

For the purpose of a theoretical evaluation, the intensity of oscillating light is converted to number of excitations per second coming to PSI and PSII, termed *kLI0* and *kLII0*, respectively, assuming a 1:1 ratio for the intensity of excitation light in μmol photons m^−2^ s^−1^ to the number of excitations per second. This is based on previous calculations (Lazár and Pospíšil, 1999). Thus, *kLI0* and *kLII0* were changing from 0 s^−1^ to 250 s^−1^.

### Measurement of the oscillations

The experimental protocol for measurement the oscillations always started by exposing a leaf for 5 s to measuring flashes to obtain optical signals in the dark-acclimated state of the plant. Subsequently, the leaf was illuminated by the actinic red light at a constant intensity of 250 μmol (photons) m^−2^ s^−1^ for 10 s, so that photosynthetic induction started under constant light illumination. After that, the light oscillations were initiated, first with irradiance decreasing from the maximum (250 μmol photons m^−2^ s^−1^) to the minimum (0 μmol photons m^−2^ s^−1^) along a cosine function. The first 60 oscillations, each *T*=1 s long, were performed in the first minute, followed by 3 min in which another 60 oscillations, were given, each *T*=3 s long. This was immediately followed by more sets of light oscillations with *T*=10, 20, 30, 60, 120, 180, and 300 s, but only five periods of each were applied. This protocol was repeated for 3–4 replicates, always using a new dark-acclimated plant. Data are presented as mean values of the signals obtained in the last two oscillation periods, during which the signals followed sustained, reproducible dynamic patterns.

An example of the measured signals is shown in Supplementary Fig. S1 presenting the amplitude of the forced oscillations in comparison to the initial magnitude of the signals caused by the illumination with the constant light intensity. In the case of P515, a drift of the signal was present. This probably reflects two phenomena: (1) an absorption change at 505 nm caused by the light-induced formation of zeaxanthin in the first few minutes of illumination (Ruban and Wilson, 2021), followed by (2) a slow absorption change at 550 nm due to red-shifted changes of carotenoid absorption under its strong excitation coupling with a Chl molecule, caused by slow conformational changes in the light-harvesting complexes of PSII where zeaxanthin was already bound (Van Wittenberghe *et al.*, 2019). We note that the drift in P515, and also in the I820 signal, was present occasionally and to different extents.

### Evaluation of quantum yields

For determining the quantum yields, a leaf from a fresh plant was first illuminated only by the measuring flashes, and measurements were taken of the minimal ChlF (*F*_0_) and minimal I830 (*P*_0_) for the dark-acclimated state. A MTSP was given to transiently reduce the acceptor side of PSII and determine the maximal ChlF (*F*_M_) characterizing the dark-acclimated state. Following a short interval of darkness, far-red light was used to oxidize the electron transport chain on the donor side of PSI, followed by another MTSP to fully oxidize its P700 primary donor and determine the maximal I830 (*P*_M_). These are standard routines for determination of the *F*_0_, *F*_M_, *P*_0_, and *P*_M_ values.

After determining these maximal and minimal values in dark-acclimated plants, the actinic red light with constant intensity of 250 μmol photons m^−2^ s^−1^ was switched on for 10 s to induce light acclimation. The oscillations in actinic light intensity were then started beginning from the maximal value, approximating the cosine function. Four-and-half oscillations with a period of 60 s each induced a largely stationary dynamic pattern, and measurement of quantum yield was then initiated by giving the first of the MTSPs at the beginning of the oscillation. The reporter signals for ChlF and I830 just before the MTSP were denoted as *F*_t_(0s/60s) and *P*_t_(0s/60s), respectively, and the signals read at the end of the MTSP were donated as *F*_M_´(0s/60s) and *P*_M_´(0s/60s), where subscript t and the first number in the brackets stand for the time from the beginning of the sinusoidal oscillation period. The second MTSP was given 63 s later in the next oscillation, phase-delayed by 3 s from the preceding one, and thus yielding signals denoted as *F*_t_(3s/60s), *F*_M_´(3s/60s), *P*_t_(3s/60s), and *P*_M_´(3s/60s). By repeating this measuring algorithm in the next 19 oscillations, we obtained signals evenly covering every 3 s of the whole oscillation period, ending with *F*_t_(60s/60s), *F*_M_´(60s/60s), *P*_t_(60s/60s), and *P*_M_´(60s/60s) from the last MTSP in the last period. Having only one MTSP for each of the 21 one-minute oscillation periods ensured that the MTSPs did not have a significant impact on the measured dynamics. This was demonstrated by the fact that the shape of the oscillations during the 21 periods were the consistently the same (Supplementary Fig. S2). This quantum yield measurement was repeated with leaves of four plants to obtain mean values of the measured parameters.

### Evaluation of partitioning of the PMF

The dynamics of the P515 signal (difference of the leaf transmittance between 550 nm and 515 nm) determined in separate experiments in which the actinic light (same intensity range as described above) was switched off at different points (phase shifts) along the actinic light oscillation period. The protocol always consisted first of nine-and-half oscillations each 60 s long to induce a stationary P515 dynamic pattern, and then during the following periods the light was turned off for 60 s at various phases of the oscillation. The P515 value just before the actinic light was turned off was denoted as *E*_t_. In the dark, the P515 signal first sharply decreased to a minimum, *E*_min_, followed by a slower increase to a maximum, *E*_max_. The actinic light was subsequently switched off with different phase-delays in 6-s steps, so that the following datasets were obtained: [*E*_t_(0/60s), *E*_min_(0/60s), *E*_max_(0/60s)], [*E*_t_(6/60s), *E*_min_(6/60s), *E*_max_(6/60s)], [*E*_t_(12/60s), *E*_min_(12/60s), *E*_max_(12/60s)], … [*E*_t_(60/60s), *E*_min_(60/60s), and *E*_max_(60/60s)]. Before each phase-delayed dark interval, the sinusoidal actinic light was on (from its maximal value) for two-and-a-half periods, which was enough to achieve the same stationary periodical changes of P515 signal again (Supplementary Fig. S3). This assay was repeated with leaves from three different plants to determine mean values of the measured parameters.

### Evaluated parameters

The primary data for ChlF and I830 obtained as described above were used to calculate characteristic quantities that can be used for direct molecular interpretation (Klughammer and Schreiber, 1994; Hendrickson *et al.*, 2004; Schreiber and Klughammer, 2008a; Lazár, 2015). The quantities are defined as follows:

- the effective quantum yield of PSI photochemistry, *Y(I)*=(*P*_M_´–*P*_t_)/(*P*_M_–*P*_0_);
- the quantum yield of non-photochemical quenching of PSI excitation energy due to a limitation on the PSI donor side, *Y(ND)*=(*P*_t_–*P*_0_)/(*P*_M_–*P*_0_);
- the quantum yield of non-photochemical quenching of PSI excitation energy due to a limitation on the PSI acceptor side, *Y(NA)*=(*P*_M_–*P*_M_´)/(*P*_M_–*P*_0_);
- the effective quantum yield of PSII photochemistry, *Y(II)*=(*F*_M_´–*F*_t_)/*F*_M_´;
- the quantum yield of constitutive non-regulatory non-photochemical quenching of PSII excitation energy, *Y(f,D)*=*F*_t_/*F*_M_;
- the quantum yield of light-induced regulatory non-photochemical quenching of PSII excitation energy, *Y(NPQ)*=(*F*_t_/*F*_M_´)–(*F*_t_/*F*_M_);
- the non-photochemical quenching parameter, *NPQ*=(*F*_M_–*F*_M_´)/*F*_M_´=*Y*(*NPQ*)/*Y*(*f,D*).

Some of the parameters above are arithmetically mutually dependent: *Y(I)*+*Y(ND)*+*Y(NA)*=1 and *Y(II)*+*Y(NPQ)*+*Y(f,D)*=1.

The coefficient of photochemical quenching of PSII excitation energy assuming energetically connected PSII units, *qCU*, the minimal ChlF for the light-acclimated state, *F*_0_´, and the effective quantum yield of alternative electron transport (AET), *Y(AET)*, were calculated from the measured values in Microsoft Excel according to Kramer *et al.* (2004), Oxborough and Baker (1997), and Yamori *et al.* (2011), respectively, as follows:

- *qCU*=(*F*_M_´–*F*_t_)/{[(*p*/(*p*–1)](*F*_t_*–F*_0_´)+*F*_M_´–*F*_0_´}, where *p*=0.55 (Joliot and Joliot, 1964) is the probability of energy transfer between the connected PSII units;
- *F*_0_´=*F*_0_/{[(*F*_M_–*F*_0_)/*F*_M_] + (*F*_0_/*F*_M_´)};
- *Y(AET)*=*Y(I)*–*Y(II)*.

The primary data for the P515 signal were used for estimating changes of PMF and its partitioning into its chemical (∆*pH*-dependent) and electrical (∆*Ψ*) components. By modifying the approach developed for continuous constant light and a steady-state (Cruz *et al.*, 2001; Schreiber and Klughammer, 2008b), we used the following formulae for the PMF and its partitioning in oscillating light::

- *PMF*=*E*_t_–*E*_min_;
- ∆*pH*-dependent part of PMF=*E*_max_–*E*_min_;
- ∆*Ψ*=*E*_t_–*E*_max_.

This approach enables evaluation of changes (in relative units) of *PMF* itself and its parts (not as fractions of *PMF*) during the course of oscillating actinic light intensity.

## Results

**Fig. 1.**
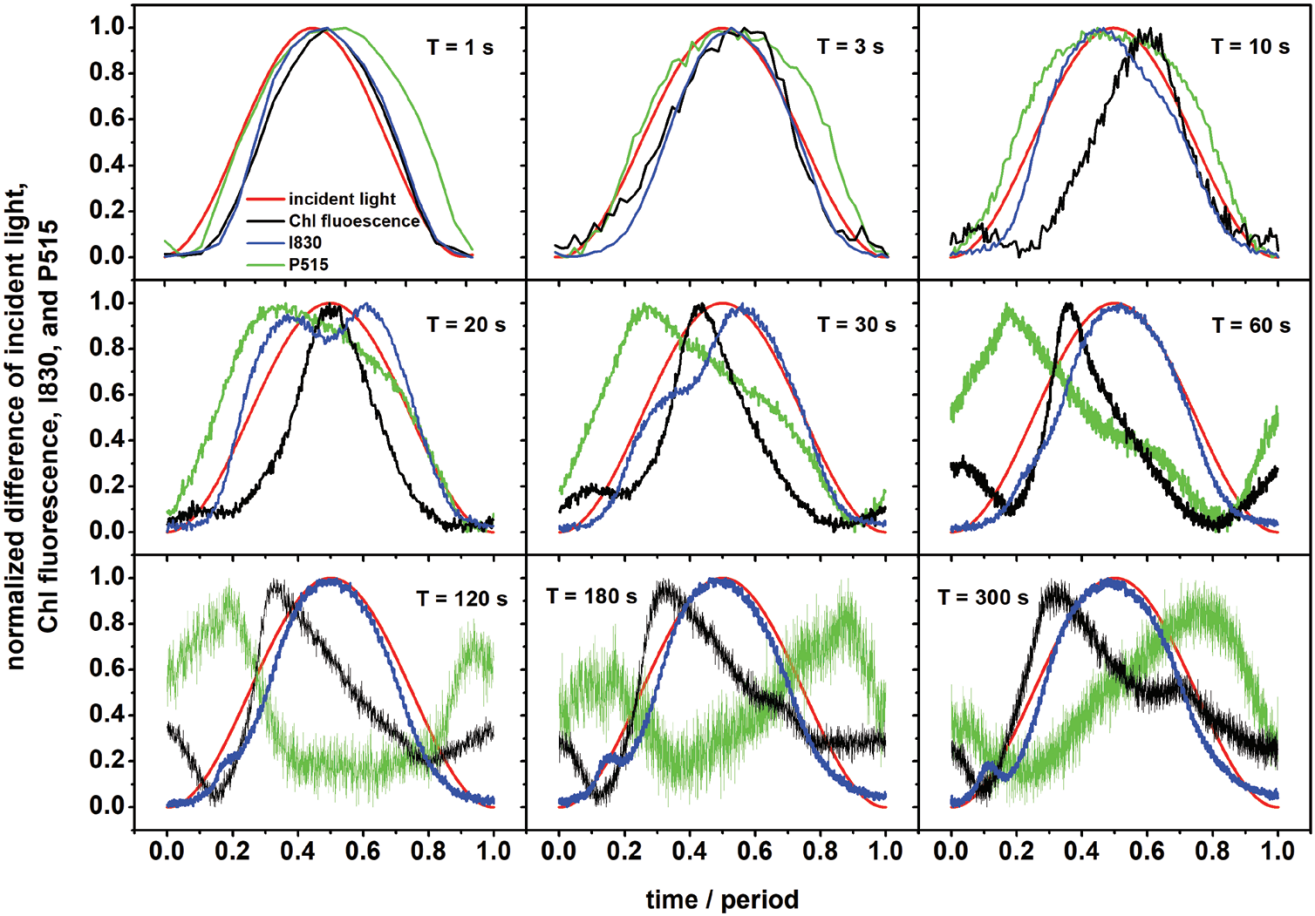
Dynamics of the normalized differences of chlorophyll (Chl) fluorescence, and I830 and P515 signals in pea leaves during exposure to oscillating light. The leaves were exposed to oscillating red light with maximal intensity of 250 µmol of photons m^−2^ s^−1^ and different periods of oscillation, *T*. Time is presented as the proportion from the beginning of the oscillation period. The data were obtained by averaging the signals in the last two oscillation periods applied for each period, in which the signals followed sustained, reproducible dynamic patterns. See Material and methods for details. For an example of data variability, see Fig. 3.

Pea leaves were exposed to red actinic light oscillating between 0–250 μmol photons m^−2^ s^−1^ with periods *T* ranging from 1 s to 300 s, and this resulted in dynamic patterns of the ChlF, I830, and P515 signals (Fig. 1). These dynamics represent stationary patterns, i.e. the shapes of the oscillations were sustained over more extended periods under the light oscillation patterns, and they were achieved after acclimation to several periods of light oscillation at each of the *T* values examined (see Material and methods). Since the sinusoidal oscillating light forced the measured signals to oscillate, we hereafter refer to the changes in the measured signals as ‘forced oscillations’. This terminology is consistent with standard textbooks (Stanford and Tanner, 1985) as well as with papers describing the phenomenon in plant research (Nedbal and Březina, 2002; Nedbal *et al*., 2003, 2005; Nedbal and Lazár, 2021).

For the two shortest periods of 1 s and 3 s, the signals closely followed the light intensity that was forcing the processes (red line in Fig. 1). The ChlF (black line) and I830 (blue line) signals responded to the increasing light in the first half of each period with a slight delay, while the rise of P515 (green line) was nearly simultaneous with that of the light. In contrast, the decline of P515 in the second half of each period followed the decline in light with a significant delay, indicating that a deviation from the course of the oscillating light in this frequency domain was more substantial for P515 than for the reporters of the primary reactions in PSII (ChlF) and PSI (I830). Activation of mechanisms leading to the rise in the P515 signal was nearly instantaneous, while deactivation occurred with a delay.

The P515 signal became convoluted as *T* increased between 20 s and 60 s, with the signal reaching its maximum when the light was still increasing. This trend led to a further differentiation as the periods further increased from *T*=120 s to *T*=300 s, resulting in a broad signal depression when the light intensity was high.

The dynamics of ChlF (Fig. 1) exhibited either two (*T*=10–120 s) or three (*T*=180–300 s) local maxima. Except *T*=10 s, the highest ChlF maximum always appeared when the light intensity was increasing. It may tentatively be assumed that this reflected a decrease in the photochemical quenching as congestion of the electron transport pathways progressed in high light. A drop in ChlF followed the highest maximum, probably due to an onset of the NPQ mechanisms. Since the position of the maximum approximately coincided with the light maximum for the periods *T*=1 s and *T*=3 s, the photochemical quenching was decreased proportionally to light intensity, and NPQ was probably not fast enough to respond. When *T*=10 s, the main ChlF maximum appeared with a delay after the light maximum, indicating a mechanism other than a trivial decrease of photochemical quenching in homogeneous PSII. With the more extended periods of the light oscillation (*T*=20–300 s), the main maximum appeared sharper and peaked at or before the maximum light was achieved. It can tentatively be presumed the onset of NPQ occurred before the slowly increasing light reached its maximum. The smaller secondary maxima of ChlF were probably due to CET or regulatory nonlinearity (see Introduction). Supporting our proposed roles of NPQ and CET, Supplementary Fig. S4 shows the forced oscillations in ChlF with a period *T*=60 s as measured with the Arabidopsis *npq4* mutant, which lacks PsbS-dependent NPQ (Li *et al.* (2000), and the Arabidopsis *pgrl1ab* mutant, which lacks the main, antimycin-A sensitive, PGR5/PGRL1-dependent CET pathway (DalCorso *et al.* (2008). However, Takagi and Miyake (2018) have suggested that processes other than CET malfunction occur in the *pgr1ab* mutant, and hence the involvement of CET in the appearance of the smaller secondary maxima in ChlF should be further explored. In addition, it can also be considered that the PSII and plastoquinone pool are heterogeneous (considered in Lazár, 2003). Hence, different kinetics of reduction of heterogeneous PSII and/or heterogeneous plastoquinone pool could contribute to the appearance of more than one maximum in ChlF for light oscillation periods of 10 s and longer.

The signal I830, which is predominantly attributable to the oxidation of the P700 primary donor in PSI, followed the light oscillation with two interesting additional dynamic features: two distinct dynamic peaks appeared around the period *T*=20 s and a shift of the peaks to shorter times together with a decrease of the first peak as the period increased. The latter lead during the periods *T*=120–300 s to the appearance of a minor, narrow secondary maximum that occurred in low light in the early rising phase. These non-linear dynamic features might reflect P700 oxidation due to transient imbalance on the donor and acceptor sides of PSI. Considering that the I830 signal is somewhat affected by the redox changes in plastocyanin and also in a minor way by ferredoxin, the observed secondary dynamic patterns might be related to these redox components to some extent. It should also not be ignored that PSI can be heterogeneous, with dynamically distinct pools in the stroma lamellae and at the edges of the grana (e.g. Albertsson, 2001). The dynamic heterogeneity might also be expressed in distinct patterns during the oscillations.

**Fig. 2.**
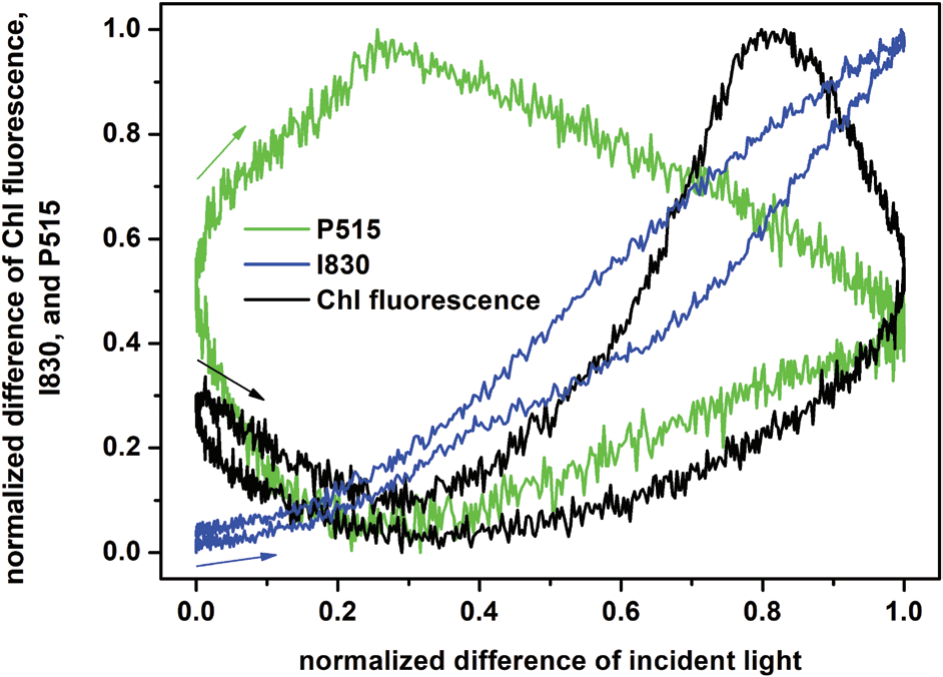
Dependence of the normalized differences of chlorophyll (Chl) fluorescence, and I830 and P515 signals in pea leaves during exposure to oscillating light on the normalized difference of the incident light intensity (input–output graph). The leaves were exposed to oscillating red light with maximal intensity of 250 µmol of photons m^−2^ s^−1^ with a period of oscillation of *T*=60 s. The graph was constructed from the data presented in Fig. 1. The position and direction of the arrows indicate the way each signal changes at the beginning of the light oscillation period.

The tentatively proposed explanations outlined above do not bring detailed insights into the dynamic phenomena and regulations that occurred in the oscillating light, and hence a more focused study was required. For this, we selected the period *T*=60 s, which is known to be characteristic of spontaneous oscillations in plants (e.g. Ferimazova *et al.*, 2002; Lazár *et al.*, 2005) and which was identified as a substantial resonance period of regulatory feedback by Nedbal and Březina (2002). First, we took the signals for *T*=60 s shown in Fig. 1 and translated them into an input–output graphical presentation shown in Fig. 2, following the approach of Nedbal *et al.* (2005). All the three measured (output) signals responded non-linearly to the input signal (incident light intensity) and showed a hysteresis: the signal dynamics in the ascending light phase differed from the descending light phase. In physics, the memory effect often explains the hysteresis, i.e. the system history influences the present state. For kinetics in a simple reversible reaction, a memory effect reflects the fact that a reaction in one direction is slower than a related reaction in the opposite direction. For example, in so-called mnemonical (or hysteretic) enzymes, their activation is much slower than their inactivation (Roussel, 1998) or, in photoprotection of photosynthesis by NPQ, epoxidation of zeaxanthin to violaxanthin is slower than de-epoxidation of violaxanthin to zeaxanthin (Matuszyńska *et al.*, 2016). In a more complex system of reactions, the memory effect reflects an interplay of all the reactions involved, including feed-forward and feed-back reactions. The strongest hysteresis was observed for P515, which confirms that it is a very integrative signal that greatly depends on past processes. A minimal hysteresis was observed for I830, reflecting the primary donor of PSI, which is largely light-driven by the primary photochemistry in the reaction centers (RCs) of PSI. The ChlF signal exhibited a complex behavior because it is a composite of primary photochemistry, the redox state of (mostly) the downstream electron transport chain, and the regulatory NPQ. CET, which is inherently non-linear, also influences the plastoquinone redox state.

**Fig. 3.**
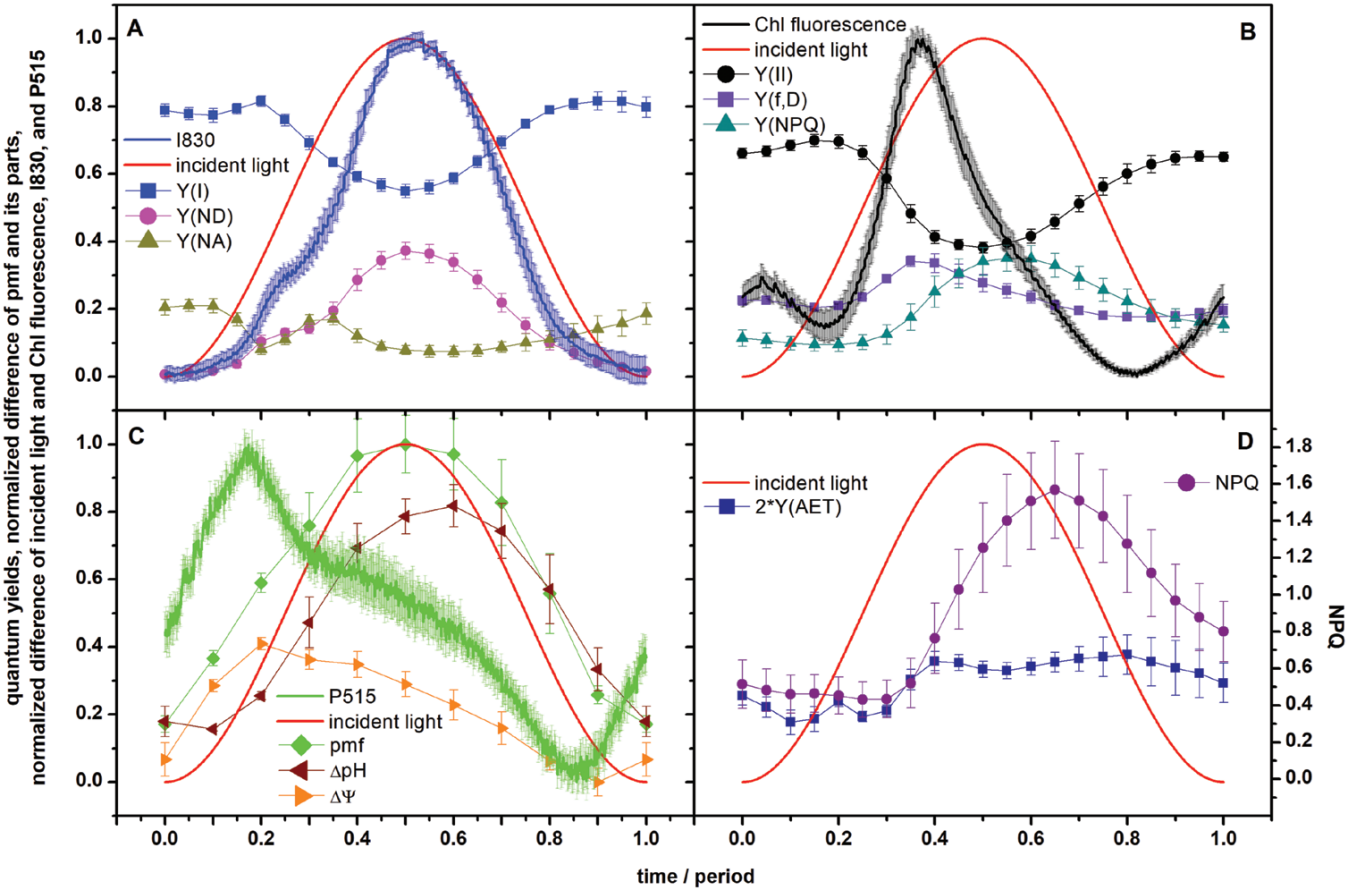
Parameters of photosynthetic energy partitioning during forced oscillations with a period of 60 s based on chlorophyll (Chl) fluorescence, and I830 and P515 signals in pea leaves. The leaves were exposed to oscillating red light with maximal intensity of 250 µmol of photons m^−2^ s^−1^. Time is presented as the proportion from the beginning of the oscillation period. See Material and methods for details. (A) The I830 signal, and the effective quantum yield of PSI photochemistry [*Y(I)*] and quantum yields of non-photochemical quenching of PSI excitation energy due to limitation at the PSI donor [*Y(ND)*] and acceptor [*Y(NA)*] sides. (B) The Chl fluorescence signal, and the effective quantum yield of PSII photochemistry [*Y(II)*] and quantum yields of constitutive non-regulatory [*Y(f,D)*] and of light-induced regulatory [*Y(NPQ)*] non-photochemical quenching of PSII excitation energy. (C) The P515 signal, and the relative proton motive force (pmf) together with its ∆*pH*-dependent and ∆*Ψ*-dependent components. (D) The effective quantum yield of alternative electron transport [*Y(AET)*] (multiplied by 2 for a better visualization) and *NPQ*. All data are means (±SD), *n*=3–4.

To separate the individual factors contributing to the dynamics seen in Fig. 2, we used multiple-turnover saturating pulses of light that transiently congested the electron transport pathways and allowed the quantum yields in both photosystems to be quantified (Fig. 3A, B). Alternatively, the oscillating actinic light was switched off for a while, which allowed quantification of *PMF* and its partitioning (Fig. 3C). It should be noted that the redox states of PSI and PSII, and the PMF and its parts can change rapidly during oscillating light. Thus, the measured PSI and PSII quantum yields and the PMF and its parts might not reflect their instantaneous states. However, we had applied the procedures mentioned above only for the cases when the actinic light oscillated with a period of 60 s. We assume that under these conditions the redox changes of PSI and PSII, and the changes in PMF and its parts caused by the oscillating actinic light are slow enough to be fully captured by the measured signals and the parameters evaluated from the signals.

The changes in the effective quantum yield of PSI photochemistry, *Y(I)*, were anti-parallel to the changes in the incident light intensity (Fig. 3A), reflecting oxidation of P700 by the intense light, which reduced the yield. This process essentially formed the prominent peak in the I830 signal. The quantum yield of quenching of PSI excitation energy due to the limitation on the acceptor side of PSI, *Y(NA)*, was strongly modulated in the ascending low-light phase, whereas it seemed to hardly change in the other phases of the oscillation. The limitation on the PSI acceptor side is the reason why the effective quantum yield of PSI photochemistry was lower than unity at the beginning and the end of the light period, and consequently that not all the RCs of PSI are open (see below) at the beginning and the end of the light period. The initial high value of *Y(NA)*, followed by a dip and a local maximum at ~0.2 and ~0.35 of the period, respectively, showed the same course as that of ChlF (Fig. 3B), suggesting that the retardation of the reactions on the acceptor side of PSI might also be involved in the changes in ChlF. An effect of the redox state of PSI on ChlF has also been reported previously (Schansker *et al.*, 2005; Lazár, 2009). The quantum yield of the PSI excitation energy quenching due to the limitation on the PSI donor side, *Y(ND)*, (Fig. 3A) also contributed to the modulation of the I830 signal around the minor peak. *Y(ND)* had a small local maximum at the minor peak position, while *Y(NA)* had a local depression in the same phase, both probably indicating a transiently reduced flow of electrons from the donor side of PSI. However, a dominant *Y(ND)* modulation came later, close to the light maximum. Changes in *Y(ND)* followed the changes in the measured I830 signal. This could be anticipated since the quenching is realized when the donor side of PSI is oxidized, and the I830 signal mainly reflects the amount of P700^+^. As noted above, the data presented in Fig. 3A might be distorted by the redox changes of plastocyanin and ferredoxin.

The quantum yields related to the function of PSII based on the measured ChlF values are shown in Fig. 3B, whilst Supplementary Fig. S5 presents quantum yields evaluated based on corrected values of *F*_M_ and *F*_M_´, which did not consider contribution of variable ChlF originating from the closed RCIIs (see Introduction and Supplementary Protocol S1). The effective quantum yield of PSII photochemistry, *Y(II)* (Fig. 3B), displayed a pattern that was roughly anti-parallel to the light oscillation only slightly delayed in phase, similar to *Y(I)* (Fig. 3A). The delay indicated memory effects in the PSII photochemistry. Memory probably also caused the asymmetry in the pattern (hysteresis); the decline of *Y(II)* when the light was increasing was steeper than the increase when the light was decreasing in the second half of the light period.

The quantum yield of regulatory NPQ of the PSII excitation energy, *Y(NPQ)* (Fig. 3B), was delayed relative to the light oscillation, forming a wide maximum in high light around the mid-period that persisted long into the descending light phase. The quantum yield of the constitutive non-regulatory NPQ of PSII excitation energy, *Y(f,D)* (Fig. 3B), was similar to the dynamics of ChlF. This could be expected since the rate constants of the constitutive non-regulatory NPQ and of the ChlF are similar, and are often joined into one value in mathematical models (Lazár, 2015). Thus, changes in *Y(f,D)* and ChlF should follow the same trend. Similarly to PSI (Fig. 3A), the constitutive [*Y(f,D)*] and the regulatory [*Y(NPQ)*] NPQ were the reasons why the effective quantum yield of PSII photochemistry [*Y(II)*] was lower than unity at the beginning and the end of the light period.

The values of *Y(II)* stayed lower than those of *Y(I)* throughout the entire illumination period. Differences between these parameters can be ascribed to alternative electron transport (AET) (e.g. Yamori *et al.*, 2011). By AET we mean all electron transport pathways except the linear one, namely PSII → plastoquinone pool → cytochrome b_6_/f → plastocyanin → PSI → ferredoxin → NADP^+^. The effective quantum yield of AET, *Y(AET)*, is shown in Fig. 3D (left axis, multiplied by 2 to visualize the changes better). The value of *Y(AET)* varied between ~0.1 and ~0.2, with the lower values in the first half of the period when the light was rising. Variation in *Y(AET)* reflects an interplay of multiple pathways (reviewed by Alric and Johnson, 2017).

The *NPQ* parameter is shown in Fig. 3D. Since *NPQ*=*Y(NPQ)*/*Y(f,D)*, it represents the ratio between the rate constants for light-induced regulatory NPQ and constitutive non-regulatory NPQ of the PSII excitation energy. The increase and decrease of *NPQ* were delayed with respect to changes in the incident light intensity. This delay in the increase reflects the time constant of activation of the regulatory NPQ of PSII excitation energy, which in this case takes ~15% of the period, i.e. about 9 s. Further, the changes in *NPQ* roughly occurred at about the same oscillation phase as those of *Y*(*AET)*. This can be understood by considering that AET also includes as a component the CET around PSI (reviewed by Alric and Johnson, 2017) and that CET acidifies the lumen, a prerequisite for the NPQ regulation (reviewed by Holzwarth and Jahns, 2014). Thus, parallel dynamics of *Y(AET)* and *NPQ* are conceivable.

By analysing the P515 signal during the forced oscillations, we obtained the relative changes of *PMF* and its ∆*pH*-dependent and ∆*Ψ* components (not as fractions of *PMF*, see Material and methods for details). The oscillating illumination changed *PMF* and the contributions of its chemical and electrical components (see Fig. 3C, which also shows the P515 signal). *PMF* approximately followed the light intensity, with apparent deviations at the beginning and end of the light period. The ∆*pH*-dependent part of *PMF* was delayed relative to the light by ~0.1 of the period (≈6 s). Since ∆*pH* is driven by the water-splitting in PSII and oxidation of plastoquinol by cytochrome (cyt) b_6_/f in one direction, and by CF_0_-CF_1_ ATP-synthase in the opposite direction, the observed delay of ~6 s might reflect the interplay of all these processes. *NPQ* (Fig. 3D) was modulated by the oscillating light in a pattern that was similar but phase-shifted to the ∆*pH*-dependent part of *PMF* (Fig. 3C). The phase delay confirms that the acidification of the lumen (reflected in ∆*pH*) is not the only driver of the light-induced NPQ (reviewed by Holzwarth and Jahns, 2014).

The dynamic patterns of ∆*Ψ* and P515 were similar, which is not surprising since the P515 signal is a measure of the electrochromic shift of pigments due to ∆*Ψ* (see Introduction). The value of ∆*Ψ* initially increased with increasing incident light intensity, with a maximum at 0.2 of the period (Fig. 3C), after which it then decreased even though the incident light intensity continued increasing. We tentatively propose that there is a flux of other positive ions (mostly K^+^ but Mg^2+^ cannot be ruled out) from the lumen to stroma and of negative ions (Cl^−^) in the opposite direction that both counteract the charge of accumulated protons in the lumen (e.g. see Cruz *et al.*, 2001; Davis *et al.*, 2017; Lyu and Lazár, 2017, 2022; Li *et al.*, 2021) and are responsible for this dynamic feature in ∆*Ψ*.

**Fig. 4.**
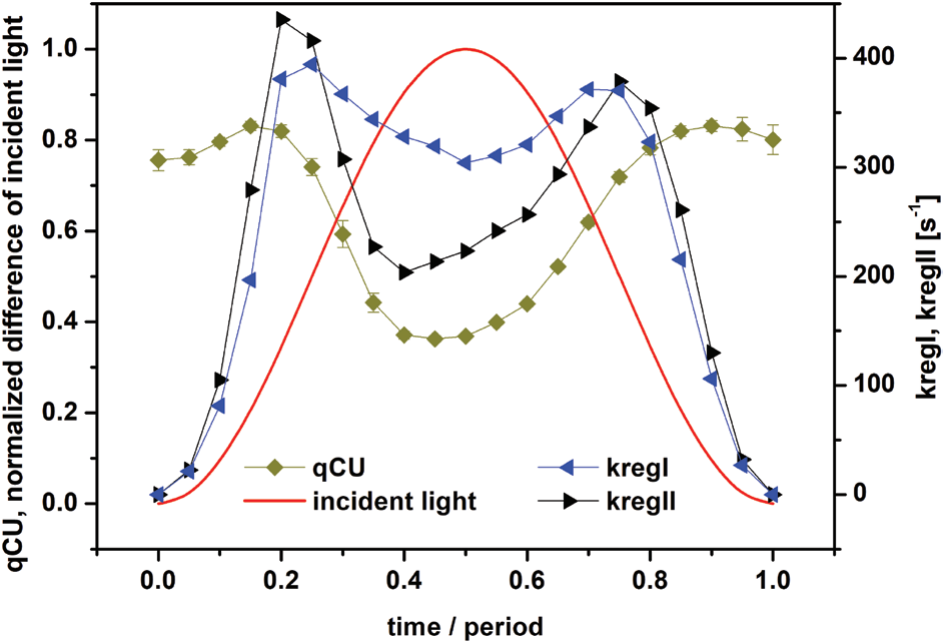
Dynamics of coefficient of photochemical quenching of PSII excitation energy (*qCU*), and of the PSI rate constant *kregI* and PSII rate constant *kregII* in pea leaves. The leaves were exposed to red light oscillating with period of 60 s with maximal intensity of 250 µmol of photons m^−2^ s^−1^. Time is presented as the proportion from the beginning of the oscillation period. See Material and methods for details. The value of *qCU* reflects the fraction of open PSII reaction centers, and *kregI* and *kregII* reflect the apparent rate constants of all regulatory mechanisms causing re-opening of PSI and PSII, respectively, during the forced oscillations. Data for *qCU* are means (±SD), *n*=4.

To gain further insights into the dynamics of the regulation, we also determined the coefficient of photochemical quenching of PSII excitation energy, *qCU*, from ChlF (Fig. 4), which reflects the fraction of open PSII RCs (RCII_o_) in the light-acclimated state, assuming energetic connectivity among PSII units (Kramer *et al.*, 2004; reviewed by Lazár, 2015). During the first quarter of the period, when the light intensity increased strongly, *qCU* only changed a little. It started dropping, indicating PSII RCs closing sharply, only shortly before the maximum light was reached. The subsequent re-opening of the PSII RCs occurred gradually with decreasing light in the second half of the period. We note that the fraction of RCII_o_ can also be estimated using *qP* or *qL* (see Kramer *et al.*, 2004; Lazár, 2015), which are used within the model of separated units (given that PSII and its antennae are energetically separated from other PSIIs and their antennae) and the lake model (particular PSIIs share excitations from all antennae without any restrictions), respectively. We found that the dynamics of *qP* and *qL* showed a similar pattern to *qCU*, but the values were different (Supplementary Fig. S6), reflecting assumed energetic communication among the PSII units.

We further wanted to quantify at which phases of the light period regulation occurs. For that purpose, we assumed that light converts the RCII_o_s to closed PSII RCs (RCII_c_s) with a rate constant *kLII* proportional to the incident light intensity. The action of NPQ, which lowers *kLII* and all other regulatory mechanisms causing apparent re-opening of RCII_c_s, is described by an apparent rate constant of PSII regulation, *kregII*. In the framework of this simple evaluation, it is the same if the regulation causes a decrease of *kLII* or is considered as an increase of *kregII*. Since *kLII* is known (see below), we can estimate *kregII* as follows:


$$\begin{array}{l} K=kLII/kregII=RCII_{c}/RCII_{o}=\left(1-RCII_{o}\right)/RCII_{o} \end{array}$$

and then by approximating RCII_o_ by *qCU* we can write:


$$\begin{array}{l} kregII=\left(kLII\times RCII_{o}\right)/\left(1-RCII_{o}\right)=\left(kLII\times qCU\right)/\left(1-qCU\right) \end{array}$$

where *K* is an equilibrium constant. This approach is based on an assumption that the system is in a steady-state equilibrium at every time of measurement. This assumption was fulfilled in our experiments since the forced oscillations were stable with a sustained pattern.

Further, since the RCIIs are assumed to be energetically connected, upon their closing *kLII* in the above equations increases in the remaining open RCIIs, which can be described as follows (Lazár *et al.*, 2001):


$$\begin{array}{l} kLII=kLII0/\left[1-\left(P\times RCII_{c}\right)\right]=kLII0/\left\{1-\left[P\times \left(1-qCU\right)\right]\right\} \end{array}$$

where *kLII0* is the number of excitations per second coming to the RCIIs and can be estimated (see Material and methods) and follows the course of incident light intensity, and *P* (=0.55; Joliot and Joliot, 1964) is the probability of energy transfer between the connected RCIIs.

Similarly, according to the theory of PSI quantum yields (Schreiber and Klughammer, 2008a), the RCs of PSI are open (RCI_o_) when the donor side of PSI is reduced and at the same time the acceptor side of PSI is oxidized. Consequently, the fraction of RCI_o_ numerically equals *Y*(*I*) (Fig. 3A). As in the case of PSII, we assume that light converts the RCI_o_s to closed PSI RCs (RCI_c_s) with a rate constant *kLI* (see below), and therefore the apparent rate constant of PSI regulation, *kregI*, can be written as:


$$\begin{array}{l} kregI=\left(kLI\times RCI_{o}\right)/\left(1-RCI_{o}\right)=\left[kLI\times Y\;\left(I\right)\right]/\left[1-Y\;\left(I\right)\right] \end{array}$$

The value of *kLI* also increases upon closure of RCIs but we assume that they are energetically separated (i.e. *P*=0), thus:


$$\begin{array}{l} kLI=kLI0/\left[1-\left(0\times RCI_{c}\right)\right]=kLI0/\left\{1-\left[0\times Y\;\left(I\right)\right]\right\}=kLI0 \end{array}$$

where *kLI0*=*kLII0* (see Material and methods).

Changes in the regulation rate constants *kregII* and *kregI* during the forced oscillations with period of 60 s are shown in Fig. 4. They both had two peaks at the same positions, at ~0.2 and ~0.8 of the period, close to the inflection points of the light modulation. The regulation buffered the variations of RCIIo (*qCU* in Fig. 4) and RCIo [*Y*(*I*) in Fig. 3A] in the initial ascending and final descending phases of the oscillating incident light intensity. Thus, the regulations try to keep both RCIIs and RCIs open, even when the change of light intensity is at its fastest at the inflection points. The RCs only close when the further light increase towards the maximum exceeds the regulation capacity [dips in *qCU* in Fig. 4 and in *Y*(*I*) in Fig. 3A at ~0.5 of the period]. Evaluation of *kregII* using *qP* (and *P*=0) for estimation of the fraction of RCII_o_ lead to the same qualitative results (Supplementary Fig. S7); the positions of the regulation maxima were the same as based on *qCU*, but the values of *kregII* were higher for *qP*. On the other hand, if *qL* (and *P*=1) was used to estimate the fraction of RCII_o_, the maxima in the dynamics of *kregII* were not so pronounced. This was caused by a combined effect of changes of *qL* (Supplementary Fig. S6) and the value of *kLII* (for *P*=1; see equations in Supplementary Protocol S1).

## Discussion

In contrast to the spontaneous oscillations studied extensively in the 1980s (reviewed by Walker, 1992; Giersch, 1994), studies reporting on forced oscillations remain rare. The conceptual background was recently newly formulated by Nedbal and Lazár (2021) with the aim of fully exploiting the potential of forced oscillations. We note that spontaneous oscillations occur only under extreme conditions: either high CO_2_ concentration or intense actinic light must be applied. In contrast, forced oscillations can be measured at ambient CO_2_ concentration and under a range of light intensities that occur in the field. Thus, forced oscillations reflect the function and regulation of photosynthesis under natural conditions.

The results reported here complement previous studies that have mainly focused on ChlF and the related parameters: *Y(PSII)*, *Y(f,D)*, *Y(NPQ)*, *NPQ*, *F*_V_´/F_M_´ (Nedbal and Březina, 2002; Nedbal *et al*., 2003, 2005; Shimakawa and Miyake, 2018; Samson *et al.*, 2019; Nedbal and Lazár, 2021), CO_2_ assimilation rate and the I830 signal (Nedbal *et al.*, 2003; Shimakawa and Miyake, 2018), and the I830-related parameters: *Y(PSI)*, *Y(ND)*, *Y(NA)* (Shimakawa and Miyake, 2018). The novelty of our study is reporting on the forced oscillations in the P515 signal (Figs 1, 2) and on the relative changes of *PMF* and its ∆*pH*-dependent part and ∆*Ψ* (Fig. 3C), both of which were determined from the P515 signal. In addition, we calculated the apparent rate constants of regulation of RCIIs and RCIs opening, *kregII* and *kregI*, respectively, as they changed in oscillating light (Fig. 4). This analysis showed that regulation stabilized the fraction of open RCs even though the light oscillated. In other words, the photosynthetic regulation acted towards stable output with fluctuating input, i.e. stable fractions of RCII_o_ and RCI_o_ in oscillating light. This conclusion has tentatively been proposed previously (Nedbal *et al.*, 2005) based on the input–output relation of ChlF in oscillating light, and is confirmed here by solid experimental evidence. A similar conclusion has recently been reached with regards to the absorption spectra of photosynthetic pigments (Arp *et al.*, 2020).

Non-photochemical quenching would be the most common candidate to explain the regulation of PSII. This was supported by the difference in ChlF that we observed between wild-type Arabidopsis and the *npq4* mutant (Supplementary Fig. S4), which lacks PsbS-dependent NPQ (Li *et al.*, 2000), with the differences being most pronounced in the second half of the light oscillation period. It also agrees with the fact that *NPQ* had a high value, but not a maximum (Fig. 3D), at the position of the second maximum of *kregII* (Fig. 4); however, *NPQ* had a low value at the position of the first maximum of *kregII*. Thus, *NPQ* alone, initiated by the accumulation of protons in the lumen as reflected in the increase of the ∆pH-dependent component of *PMF* (Fig. 3C), cannot explain all the changes in *kregII*. On the other hand, the position of the first maximum of *kregII* and of *kregI* (Fig. 4) was the same as the position of the maximum of ∆*Ψ* (Fig. 3C). ∆*Ψ* has been reported to promote charge recombinations in PSII (e.g. Dau and Sauer, 1992; Davis *et al.*, 2016), thus causing opening of RCIIs, i.e. a decrease of ChlF. We found that ChlF slightly decreased (Fig. 3B) at the position of the first maximum of *kregII* (Fig. 4). Hence, we suggest a role of ∆*Ψ* in regulation at least of PSII in the rising phase of sinusoidal illumination. The role of ion fluxes in fluctuating light has been reported previously (e.g. Duan *et al.*, 2016; Li *et al.*, 2021). The roles of the ∆*Ψ* and ∆*pH*-dependent components of PMF on PSII regulation at different parts of the oscillating light period as noted above agree with the role of the PMF partitioning in the regulation (Avenson, *et al.*, 2004; Takizawa *et al.*, 2007; Davis *et al.*, 2017).

The CET from PSI back to the plastoquinone pool has two pathways, one sensitive to antimycin A and the other insensitive. The PGR5/PGRL1 complex (e.g. Munekage *et al.*, 2002) and the NAD(P)H-dehydrogenase-like complex (e.g. Yamamoto *et al.*, 2011) are involved in the sensitive and insensitive pathways, respectively. The CET is often considered a regulatory mechanism for electron transport in the thylakoid membrane (reviewed by Alric and Johnson, 2017) and its protective role under fluctuating light has also been reported, especially of the PGR5/PGRL1-dependend CET (e.g. Yamori *et al.*, 2016; Yamamoto and Shikanai, 2019). The CET also promotes so-called photosynthetic control, i.e. a decrease of the rate of reduced plastoquinone oxidation at cyt b_6_/f due to the accumulation of protons in the lumen (e.g. Johnson and Berry, 2021), this effect also being known as backpressure of protons (Siggel, 1976). However, we can exclude CET as the origin of the peaks in the dynamics of *kregII* and *kregI*, since it would partly re-open closed RCIs, compared to the case without any CET. However, by the reduction of the plastoquinone pool, which is also promoted by photosynthetic control, the CET would contribute to increased closure of RCIIs, which is against simultaneous changes in the same direction of RCII_o_ (*qCU* in Fig. 4) and of RCIo [*Y(I)* in Fig. 3A). As mentioned above, the regulation was maximal at low excitation light intensities. Walker *et al.* (2014) inferred that some AET pathways, for example the malate valve and Mehler reaction, were functional only at low light intensities. The Mehler reaction has been suggested to play an essential role in the photoprotection of PSI under fluctuating light (Sun *et al.*, 2020). In our case, the electron flow from PSI to malate and/or oxygen would re-open PSI and consequently also PSII, which agrees with the simultaneous changes in the same direction of RCIIo and of RCIo and consequently of *kregII* and *kregI*. Thus, we tentatively assign the regulation to the function of a part of AET. Indeed, there is a small peak in *Y(AET)* at 0.2 of the period and a broad peak at 0.8 of the period (Fig. 3D), i.e. at the exact times as the maxima of *kregII* and *kregI* (Fig. 4). Thus, a part of AET, the CET around PSI, contributes to the regulation of the NPQ, and another part of AET, probably the malate valve and/or Mehler reaction, contributes to the regulation of RCs opening. In PSII, the NPQ and the ∆*Ψ* -promoted charge recombinations (see above) might additionally contribute to keeping the RCIIs open.

The above discussion is for the data that we obtained using standard measurements and evaluation. However, as mentioned in the Introduction, RCII_c_ can also emit variable ChlF, with the effect playing a role when saturating pulses are applied, as was the case in our study. Hence, we also performed an evaluation that employed a correction of the ChlF values obtained upon saturating pulses (the *F*_M_ and *F*_M_´ values). The Supplementary Protocol S1 describes the correction, and Supplementary Figs S5, S8, and S9 show the results. This evaluation led to changes in the absolute values of related parameters but their qualitative changes during the oscillating light period were the same as in the standard evaluation, and hence they did not alter the conclusions. However, a further evaluation using the Walz DUAL-KLAS-NIR instrument based on the corrected ChlF values together with discrimination of the components contributing to the I820 signal (P700^+^, oxidized plastocyanin, reduced ferredoxin) might bring new conclusions. This will be the subject of our future work.

In this study, we mainly used wild-type plants that do not lack or overexpress particular protein(s) involved in the regulation of photosynthesis. We combined measurement of the ChlF, I830, and P515 reporter signals and evaluated related parameters describing energy partitioning in PSI, PSII, and PMF. This allowed us to infer the mechanisms of regulation of photosynthesis in fluctuating light, in our case sinusoidal. We have shown some results from mutant plants subjected to forced oscillations in our Supplementary data and we intend to present more details in a future paper. Our work shows a high potential of forced oscillations in studying the function and regulation of photosynthesis; however, as it is clear from the discussion, it is a highly complex subject. We expect further progress in understanding the forced oscillations and associated regulations from experiments involving chemical interventions by electron acceptors, donors, and inhibitors, and involving mutants that are affected in well-defined regulatory mechanisms or pathways. This will stimulate examination and further development of structure–function-based mathematical models considering particular regulatory mechanisms. Deciphering the forced oscillations will also be supported by systems identification and systems control tools that have already been successfully applied to solve homologous challenges in engineering (e.g. Schrangl *et al.*, 2020) and medicine (e.g. Doyle, 2016).

## Supplementary data

The following supplementary data are available at *JXB* online.

Protocol S1. Correction of *F*_M_ and *F*_M_´ and related evaluations.

Fig. S1. Example of raw data of typical measurements of the forced oscillations.

Fig. S2. Example of raw data of typical measurements of PSI and PSII quantum yields using the saturation pulse method.

Fig. S3. Example of raw data of typical measurement of the partitioning of PMF into its ∆*pH*- and ∆*Ψ*-dependent parts.

Fig. S4. Example of raw data of typical measurements of the forced ChlF oscillations with Arabidopsis wild-type and photosynthesis mutants.

Fig. S5. Quantum yields of PSII after the correction of *F*_M_ and *F*_M_´ values.

Fig. S6. Coefficients of photochemical quenching of excitation energy of PSII, *qP*, *qCU*, and *qL*.

Fig. S7. The rate constant *kregII* calculated based on *qP*, *qCU*, and *qL*.

Fig. S8. Coefficients of photochemical quenching of excitation energy of PSII, *qP*, *qCU*, and *qL* after the correction of *F*_M_ and *F*_M_´ values.

Fig. S9. The rate constant *kregII* calculated based on *qP*, *qCU*, and *qL* after the correction of *F*_M_ and *F*_M_´ values.

erac283_suppl_Supplementary_Protocol_S1_Figures_S1-S9

## Data Availability

The data supporting the findings of this study are available from the corresponding author, Dušan Lazár, upon request.

## Abbreviations

CET: cyclic electron transport
Chl: chlorophyll
ChlF: chlorophyll fluorescence
∆pH: difference of pH across thylakoid membrane
∆*Ψ*: difference of electric potential across thylakoid membrane, the membrane potential
MTSP: multiple-turnover saturating pulse
NPQ: non-photochemical quenching
PMF: proton motive force
RC: reaction center
TM: thylakoid membrane
