# Supplementary material for: Insights on the regulation of photosynthesis in pea leaves exposed to oscillating light
Source: J Exp Bot. 2022 Aug 29;73(18):6380–93. doi: 10.1093/jxb/erac283 (PMC9578350; doi:10.1093/jxb/erac283)
Supplement: erac283_suppl_Supplementary_Protocol_S1_Figures_S1-S9 [file erac283_suppl_supplementary_protocol_s1_figures_s1-s9.pdf]

Supplementary data

to

On the regulation of photosynthesis in pea leaves exposed to oscillating light

Dušan Lazár[1], Yuxi Niu[2], Ladislav Nedbal[1]

[1] Department of Biophysics, Faculty of Science, Palacký University, Šlechtitelů 27, 783 71 Olomouc, Czech Republic.

[2] Institute of Bio- and Geosciences/Plant Sciences (IBG-2), Forschungszentrum Jülich, Wilhelm-Johnen-Straße, D-52428 Jülich, Germany

Supplementary protocol S1. Correction of F_M and $F_M´$ and related evaluations.

Data presented in Supplementary Figs. S5, S8, and S9 represent evaluation of data presented in Fig. 3B of the main text and in Supplementary Figs. S6 and S7, respectively, based a correction of F_M and $F_M´$ whose measured values might be overestimated by variable ChlF of closed reaction centers of PSII (Vredenberg and Bulychev, 2002; Magyar et al., 2018; Laisk and Oja, 2020; Sipka et al., 2021). Thus, instead of the measured F_M and $F_M´$, we used corrected F_M and $F_M´$ values. Data presented in Laisk and Oja (2020) show that about 1/3 of ChlF measured with multiple turnover saturating light pulse (F_M and $F_M´$), as it is used in our measurements, is caused by increase of ChlF of already closed reaction centers of PSII. Thus, we considered 2/3 of the measured F_M and $F_M´$ as the corrected values of F_M and $F_M´$. We then used these corrected values for evaluation of the quantum yields of function of PSII (Supplementary Fig. S5) and of the coefficients of the photochemical quenching of ChlF, *qP*, *qCU*, and *qL* (Supplementary Fig. S8). Definition of *qCU* is provided in the main text and *qP* and *qL* were calculated as follows (Kramer et al., 2004; Lazár, 2015):

$qP = (F_M´ - Ft)/(F_M´ - F_0´)$

$qL = qP * (F_0´ / Ft)$,

where the ChlF values have the meaning defined in the main text.

When evaluating *kregII* using *qP* (and *p* = 0) or *qL* (and *p* = 1) (Supplementary Figs. S7 and S9), we used following equations:

$$kregII = (kLII * \mathrm{RCIIo})/(1 - \mathrm{RCIIo}) = (kLII * qP)/(1 - qP)$$

with

$$kLII = kLII0/(1 - (\mathrm{p} * \mathrm{RCIIc})) = kLII0/(1 - (0 * (1 - qP))) = kLII0$$

for the case of *qP*, and:

$$kregII = (kLII * \mathrm{RCIIo})/(1 - \mathrm{RCIIo}) = (kLII * qL)/(1 - qL)$$

with

$$kLII = kLII0/(1 - (\mathrm{p} * \mathrm{RCIIc})) = kLII0/(1 - (1 * (1 - qL))) = kLII0/qL$$

for the case of *qL*.

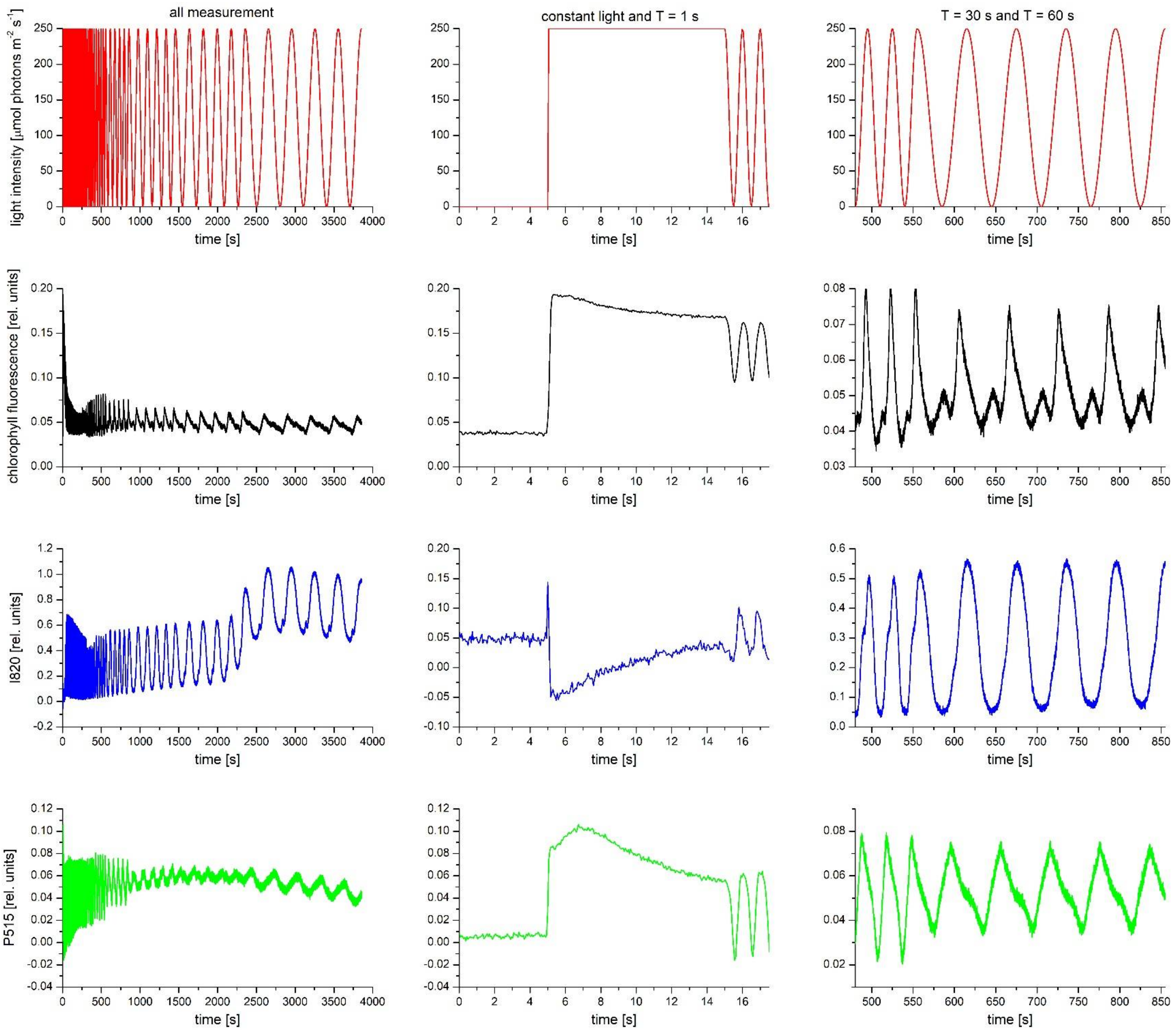
all measurement
constant light and T = 1 s
T = 30 s and T = 60 s
light intensity [µmol photons m⁻² s⁻¹]
chlorophyll fluorescence [rel. units]
I820 [rel. units]
P515 [rel. units]
time [s]

Supplementary figure S1. Example of raw data of typical measurements of the forced oscillations.

The figure shows typical measurements of oscillations of ChlF (second row), I820 (third row), and P515 (fourth tow) forced by sinusoidally oscillating incident light (first row). The first column shows the whole measurement; it mainly serves to show a drift of the baselines in the I820 a P515 signals (see Material and methods). The second and third columns show in detail selected time intervals of the whole measurement. It shows how the constant light was changed to the oscillating light with period *T* of 1 s (two and half 1-s-periods are shown; second column), and last two and half periods of 30 s changed to five periods of 60 s (third column). The data also show that shape of particular oscillations for given period of forcing has not changed during time. Four such whole measurements have been done, each with different leaf from different plant. The data presented in the Fig. 1 in the main text have been obtained by averaging two last whole periods.

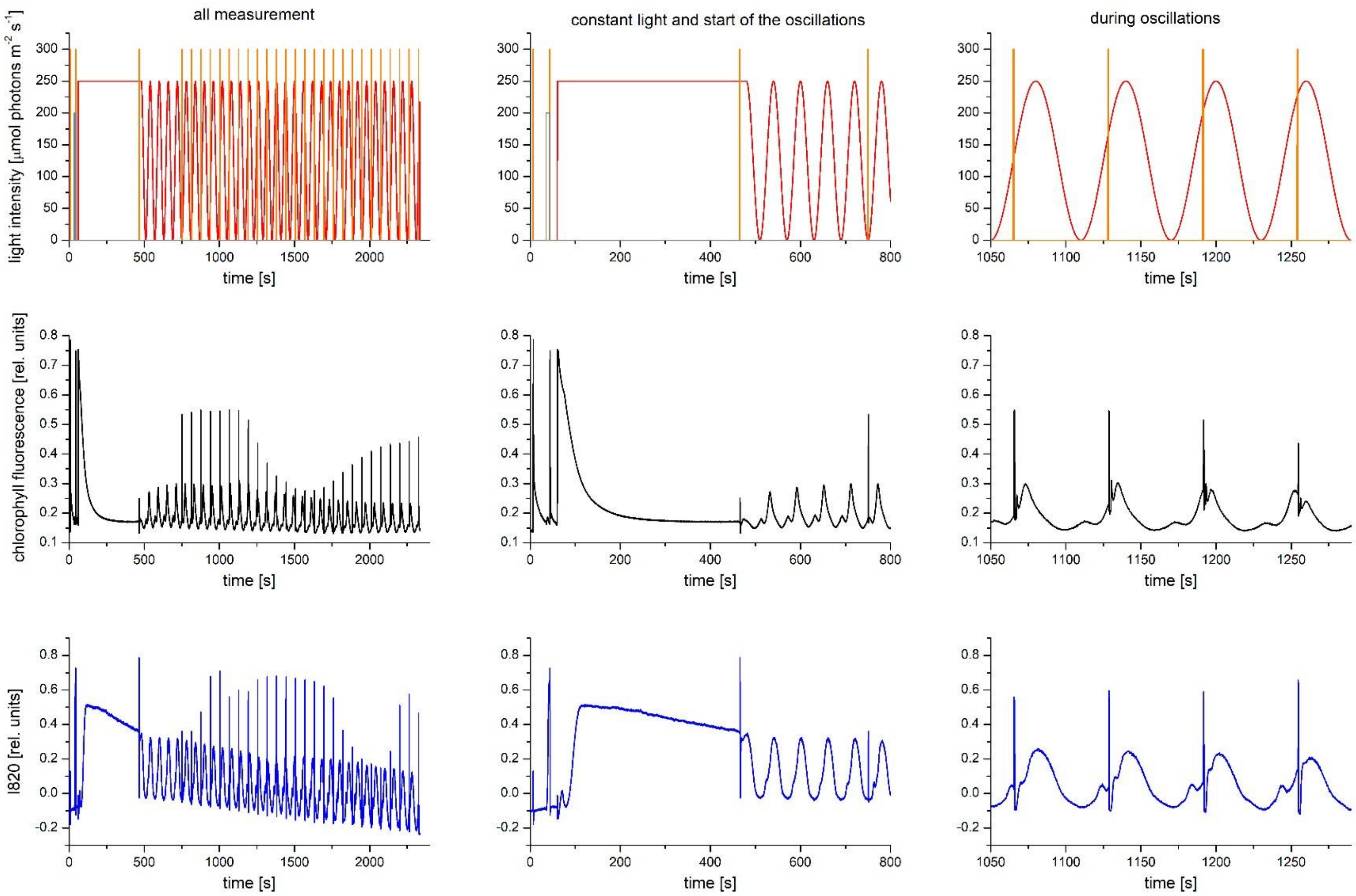

Supplementary figure S2. Example of raw data of typical measurements of PSI and PSII quantum yields using the saturation pulse method.

The figure shows typical measurements of quantum yields of PSII and PSI during oscillations of ChlF (second row) and I820 (third row) signal, respectively, forced by sinusoidally oscillating incident light (first row) with period *T* = 60 s. The grey and orange vertical lines show positions of the far-red light illumination (necessary to determine P_M) and of the MTSPs, respectively (see Material and methods), and the heights of the lines are not in scale with respect to intensity of the oscillating light. The first column shows the whole measurement; it serves to show a small drift of the baseline in the I820 signal, which does not affect evaluation of the quantum yields, and to show magnitudes of the ChlF and I820 signal caused by the MTSPs compared to the magnitude of the oscillation itself. The second and third columns show in detail selected time intervals of the whole measurement. Namely, the second column shows the first five and half oscillation periods after the constant light illumination; it demonstrates that three periods were enough to obtain the same shape of the oscillations over time. The third column shows shift of the positions of the MTSPs by 3 s at each oscillation period. Thus, (T/3) + 1 = (60/3) + 1 = 21 oscillation periods/MTSPs were necessary to measure the yields with 3-s-step during the whole 60-s-period. The same shape of the oscillations over whole measurement

show that one MTSP applied during one oscillation period had no effect on the shape of following oscillations. Three such whole measurements have been done, each with different leaf from different plant. The data presented in the Fig. 3A,B in the main text were obtained by merging responses to the shifted MTSPs in 21 oscillation periods to one oscillation period.

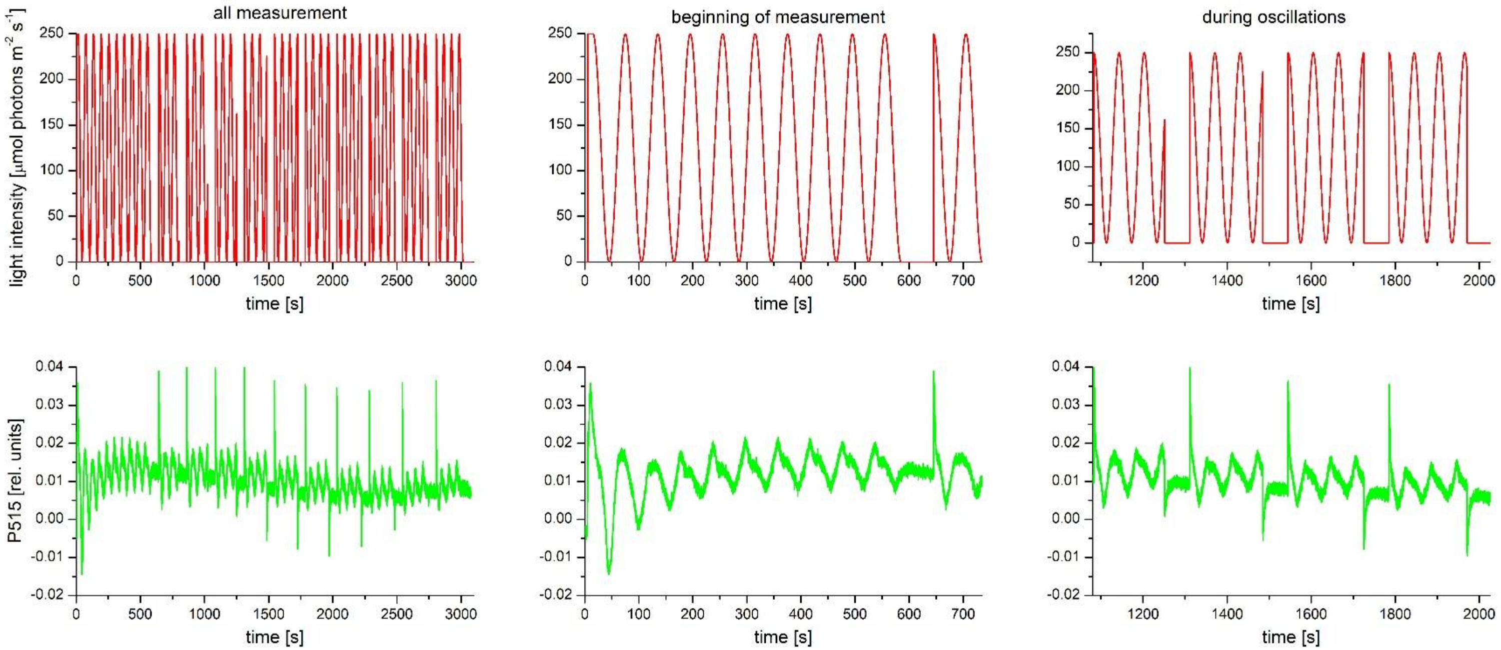

Supplementary figure S3. Example of raw data of typical measurement of the *pmf* partitioning into its ΔpH- and $\Delta\Psi$-dependent parts.

The figure shows typical measurement of the *pmf* partitioning into its ΔpH- and $\Delta\Psi$-dependent parts during oscillations of P515 signal (second row) forced by sinusoidally oscillating incident light (first row) with period T = 60 s. The partitioning was determined by switching off the light for 60 s at different phases of the oscillating light (for details, see Material and methods). The first column shows the whole measurement; it serves to show a small drift of the baseline in the P515 signal, which does not affect evaluation of the partitioning. The second and third columns show in detail selected time intervals of the whole measurement. Namely, the second column shows the first nine and half oscillation periods after 10-s constant light illumination; it demonstrates dynamics to reaching a stationary oscillatory pattern in P515 signal. The first 60-s dark interval, which started just in the beginning of the 60-s oscillating light, followed by one and half 60-s light periods are also shown. The third column shows four 60-s dark intervals each subsequently shifted 6 s forward in the oscillating light period (for details, see Material and methods). Thus, $(T/6) + 1 = (60/6) + 1 = 11$ 60-s dark intervals were necessary to measure the partitioning with 6-s-step during the whole 60-s-period. The same shape of the oscillations before beginning of the 60-s dark interval over whole measurement show that two and half light periods following the 60-s dark interval were enough to obtain stationary oscillatory pattern. Three such whole measurements have been done, each with different leaf from different plant. The data presented in the Fig. 3C in the main text have been obtained by merging responses to the shifted 60-s dark interval in 11 oscillation periods to one oscillation period.

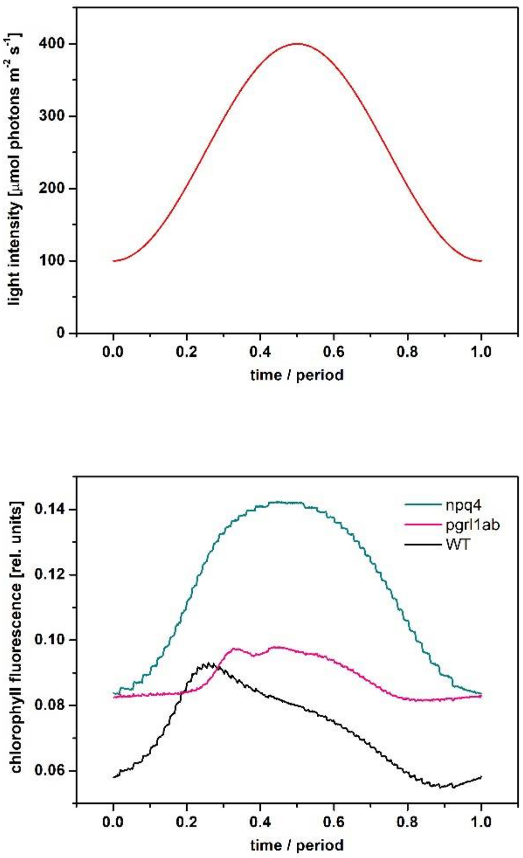

Supplementary figure S4. Example of raw data of typical measurements of the forced ChlF oscillations with wild type and mutants of Arabidopsis.

The figure shows raw data of typical measurements of ChlF forced by sinusoidally oscillating incident light with period T = 60 s. The measurements have been done with wild type and npq4 (lacks PsbS-dependent npq; Li et al., 2000) and pgrl1ab (lacks the main, antimycin-A sensitive, PGR5/PGRL1-dependent CET pathway; DalCorso et al., 2008) Arabidopsis mutants for light intensity oscillating between 100 and 400 μmol photons m^{-2} s^{-1}. The shape of the ChlF oscillation of the wild type is qualitatively similar to the shape of the oscillation with pea for the same period. The data show that ChlF of the npq4 mutant almost copies the shape of oscillation of the light intensity, suggesting a role of the npq in decrease of ChlF after its maximum observed in the wild type and in pea. On the other hand, ChlF of the pgrl1ab mutant is unchanged in the first approx. 12 seconds when the light intensity is quickly growing, suggesting a role of the CET in the initial increase of ChlF in wild type and in the small secondary maximum of ChlF in pea.

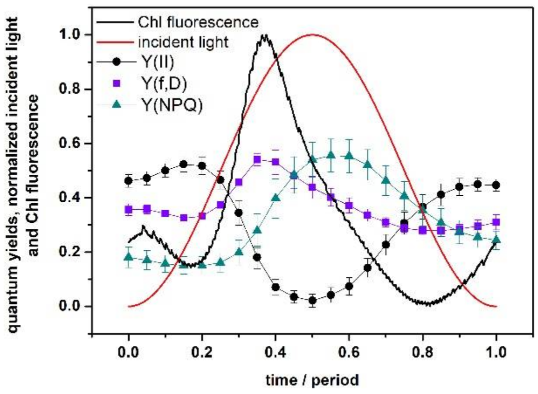

Supplementary figure S5. The PSII quantum yields after the correction of the F_M and $F_M{'}$ values.

The figure show the parameters of photosynthetic energy partitioning during the forced oscillations with period of 60 s based on ChlF, namely effective quantum yield of PSII photochemistry (*Y(II)*) and quantum yields of constitutive non-regulatory (*Y(f,D)*) and of light-induced regulatory (*Y(NPQ)*) non-photochemical quenching of PSII excitation energy. The quantum yields were evaluated considering a correction of the F_M and $F_M{'}$ values (see beginning of the supplementary data), whose measured values might be overestimated by variable ChlF of closed reaction centers of PSII. Courses of the normalized incident light and of ChlF are also shown. The symbols represent mean values (n = 4) and the error bars (sometimes hidden by the symbols) show standard deviations.

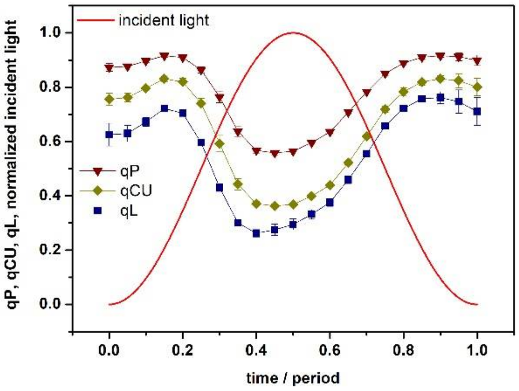

Supplementary figure S6. The coefficients of photochemical quenching of excitation energy of PSII, *qP*, *qCU*, and *qL*.

The figure shows the coefficients of photochemical quenching of excitation energy of PSII, *qP*, *qCU*, and *qL*, during the forced oscillations with period of 60 s. Course of normalized incident light intensity is also shown. All the coefficients can be used for estimation of fraction of the open reaction centers of PSII but assuming different energetic “communication” among PSII units; the units are energetically separated (*qP*), are energetically connected with a restriction (*qCU*), or are energetically connected without any restriction (*qL*). The symbols represent mean values (n = 4) and the error bars (sometimes hidden by the symbols) show standard deviations.

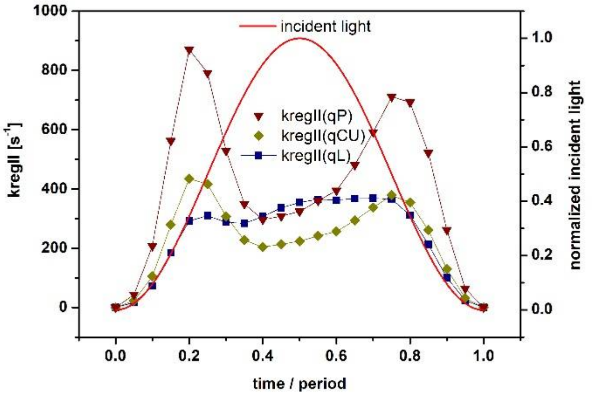

Supplementary figure S7. The rate constant *kregII* calculated based on *qP*, *qCU*, and *qL*.

The figure shows the rate constant *kregII* during the forced oscillations with period of 60 s. Course of normalized indent light is also shown. The *kregII* reflects apparent rate constant of all regulatory mechanisms causing re-opening of photosystem II reaction centers. Values of *kregII* were calculated from courses of *qP*, *qCU*, and *qL* shown in Supplementary Fig. S6.

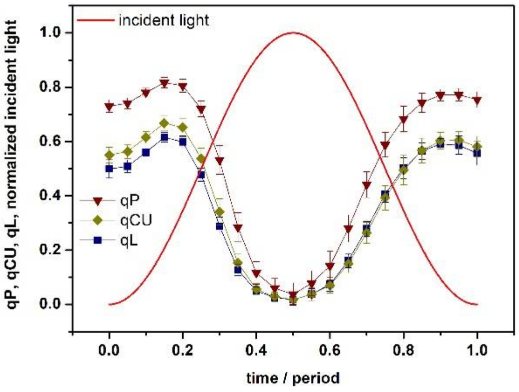

Supplementary figure S8. The coefficients of photochemical quenching of excitation energy of PSII, *qP*, *qCU*, and *qL* after the correction of the F_M and F_M' values.

The figures shows the coefficients of photochemical quenching of excitation energy of PSII, *qP*, *qCU*, and *qL*, during the forced oscillations with period of 60 s. Course of normalized incident light intensity is also shown. All the coefficients can be used for estimation of fraction of the open reaction centers of photosystem II but assuming different energetic "communication" among PSII units; the units are energetically separated (*qP*), are energetically connected with a restriction (*qCU*), or are energetically connected without any restriction (*qL*). The coefficients were evaluated considering a correction of the F_M and F_M' values (see beginning of the supplementary data), whose measured values might be overestimated by variable ChlF of closed reaction centers of PSII. The symbols represent mean values (n = 4) and the error bars (sometimes hidden by the symbols) show standard deviations.

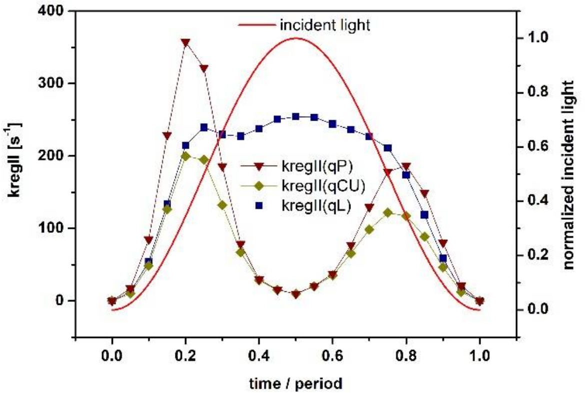

Supplementary figure S9. The rate constant *kregII* calculated based on *qP*, *qCU*, and *qL* after the correction of the F_M and $F_M{}'$ values.

The figures shows the rate constant *kregII* during the forced oscillations with period of 60 s. Course of normalized indent light is also shown. The *kregII* reflects apparent rate constant of all regulatory mechanisms causing re-opening of PSII reaction centers. Values of *kregII* were calculated from courses of *qP*, *qCU*, and *qL* shown in Supplementary Fig. S8.

References of the supplementary data

DalCorso G, Pesaresi P, Masiero S, Aseeva E, Schünemann D, Finazzi G, Joliot P, Barbato R, Leister D. 2008. A complex containing PGRL1 and PGR5 is involved in the switch between linear and cyclic electron flow in Arabidopsis. Cell 132, 273–285.

Kramer DM, Johnson G, Kiirats O, Edwards GE. 2004. New fluorescence parameters for the determination of Q_A redox state and excitation energy fluxes. Photosynthesis Research 79, 209–218.

Laisk A, Oja V. 2020. Variable fluorescence of closed photochemical reaction centers. Photosynthesis Research 143, 335–346.

Lazár D. 2015. Parameters of photosynthetic energy partitioning. Journal of Plant Physiology 175, 131–147.

Li XP, Björkman O, Shih C, Grossman AR, Rosenquist M, Jansson S, Niyogi KK. 2000. A pigment-binding protein essential for regulation of photosynthetic light harvesting. Nature 403, 391–395.

Magyar M, Sipka G, Kovács L, Ughy B, Zhu Q, Han G, Špunda V, Lambrev PH, Shen JR, Garab G. 2018. Rate-limiting steps in the dark-to-light transition of Photosystem II - revealed by chlorophyll-*a* fluorescence induction. Scientific Reports 8, 2755.

Sipka G, Magyar M, Mezzetti A, Akhtar P, Zhu Q, Xiao Y, Han G, Santabarbara S, Shen J-R, Lambrev PH, Garab G. 2021. Light-adapted charge-separated state of photosystem II: structural and functional dynamics of the closed reaction center. Plant Cell 33, 1286–1302.

Vredenberg WJ, Bulychev AA. 2002. Photo-electrochemical control of photosystem II chlorophyll fluorescence in vivo. Bioelectrochemistry 57, 123–128.
